# High-sensitivity C-reactive protein and 6-month all-cause mortality in Chinese heart failure patients

**DOI:** 10.1038/s41598-025-18265-7

**Published:** 2025-09-25

**Authors:** Ailing Zhu, Jinfeng Zhang, Shuanglin Zhou, Dehai Ge, Xiujian Zhang, Manman Hu, Zhengyong Guo, Junjun Liu

**Affiliations:** 1 Nanjing Meishan Hospital, Nanjing, 210041 People’s Republic of China; 2 Department of Psychiatry, Nanjing Meishan Hospital, Nanjing, 210041 People’s Republic of China

**Keywords:** hs-CRP, Heart failure, All-cause mortality, Linear, Association, Cardiovascular biology, Prognostic markers

## Abstract

Heart failure (HF) is a principal cause of both morbidity and mortality throughout the world. As a marker of systemic inflammation, High - Sensitivity C - Reactive Protein (hs - CRP) is elevated in HF patients and is in relation with adverse clinical outcomes. However, the relationship between hs-CRP levels and mortality of HF patients, particularly in the Chinese population, remains incompletely characterized. In this retrospective cohort research, data from a prospective HF registry involving 941 patients who were hospitalized to the Zigong Fourth People’s Hospital in Sichuan Province during January 2016 and December 2019 were analyzed. The primary endpoint was all-cause mortality within six months of admission. Because hs-CRP data did not follow a normal distribution, logarithmic transformation was carried out, and the values were stratified into tertiles to make the comparative analysis more convenient. Multivariable logistic regression, with adjustments made for potential confounding factors, was used to assess the independent prognostic value of log(hs-CRP). Constrained cubic spline transformations were used to investigate the potential nonlinear relationships between log(hs-CRP) and the risk of mortality. Among 941 selected participants, the six-month all-cause mortality rate was 3.08% (29/941). After adjusting for potential confounders, logistic regression analysis showed a significant positive association between elevated hs-CRP concentrations and increased mortality risk (OR = 2.073; 95% CI: 1.009–4.256; *p* = 0.047). Receiver operating characteristic curve analysis confirmed that hs-CRP has predictive value for mortality (AUC:0.66, 95%CI: 0.56–0.76), with an optimal cutoff value of 1.64. However, nonlinear association was not detected between these variables (log-rank *p* > 0.05). The study revealed a significant direct correlation between hs-CRP levels and six-month all-cause mortality in Chinese HF patients, suggesting that the biomarker may serve as a valuable prognostic indicator. These findings support the incorporation of hs-CRP measurement into routine evaluation protocols for heart failure patients to enhance risk stratification and guide therapeutic decision-making.

## Introduction

Heart failure (HF) represents a prevalent cardiovascular condition with rising global incidence and prevalence, imposing a significant health burden and escalating social and economic costs^1^. In Europe, HF affects approximately 3 per 1000 person-years across all age groups, with a notably higher incidence of 5 per 1000 person-years among adults^2^. China reports a weighted prevalence of 1.3% in individuals aged 35 years and older, representing over 13.7 million HF patients^3^. Despite therapeutic advancements, persistently high incidence of mortality and readmission continue to strain healthcare systems and compromise patient outcomes^2^. Consequently, there is an urgent need to identify high-risk patients with a strong tendency towards frequent readmissions and mortality, develop appropriate methods to reduce readmission and mortality rates, and prevent heart failure decompensation.

High-sensitivity C-reactive protein (hs-CRP) is the most appreciated acute phase protein and one of the most reliable indicators of inflammation, demonstrating significant elevation during inflammatory responses^4^. This biomarker is closely associated with both acute and chronic heart failure, playing a substantive role in disease initiation, progression, and development of complications. Consequently, elevated hs-CRP concentration is regarded a marker of unfavorable prognosis in heart failure patients^5^.

Multiple research findings indicate that a chronic low-grade inflammatory environment exerts sustained effects on cardiomyocyte homeostasis, contributing to persistent manifestations and progressive deterioration of chronic heart failure (CHF) and while simultaneously precipitating recurrent episodes of acute decompensation^6,7^. Current epidemiological evidence consistently demonstrates a well-established link occurring between elevated hs-CRP levels and heightened risks of adverse cardiovascular incidents, particularly increased hospital mortality rates among HF populations^8–13^. However, recent findings from Segura-Saldaña et al.^14^ suggest a paradoxical dissociation wherein, despite correlating with disease progression, hs-CRP fails to retain independent prognostic significance for mortality prediction. This observation highlights a critical knowledge gap concerning the prognostic precision of hs-CRP specifically in HF cohorts. Notably, the majority of existing evidence originates from Western populations, while data on Chinese patients remain particularly limited. Furthermore, existing studies have not systematically investigated potential nonlinear relationships between hs-CRP levels and clinical outcomes^10–12^. Such methodological limitations carry important implications for biomarker-driven risk stratification strategies, as failure to account for potential nonlinear relationships may significantly undermine the predictive accuracy of future CRP-based prognostic models for HF^15^. To address these gaps, we conducted a retrospective study to determine whether hs-CRP independently correlates with all-cause mortality in HF patients and explored potential nonlinear relationships between these variables.

## Methods

### Data origins

The research sample was sourced from an open-access, web-based database of HF patients in China, which was accessible through the PhysioNet platform^16^. In total, 2,008 in hospital patients were recruited at the Zigong Fourth People’s Hospital from December 2016 to June 2019. The specific research design as well as the details of the database have been reported previously^17^. The study plan and methods received approval from the Ethics Committee of the Zigong Fourth People’s Hospital (approval number: 2020–010). Every procedure was carried out in line with the principles set forth in the Declaration of Helsinki.

### Study sample

The study involved patients admitted with a HF diagnosis. We reviewed the electronic health records of successive patients diagnosed with HF^17^. The database covered all HF types according to the 2016 European Society of Cardiology (ESC) criteria^18^.

#### Inclusion criteria

Clinically, patients typically exhibit symptoms such as fatigue, diminished exercise tolerance, delayed post-exercise recovery, peripheral edema (particularly involving the ankles), and dyspnea (notably orthopnea and paroxysmal nocturnal dyspnea). Concurrently, physical examination may identify pathological signs including the auscultation of a third heart sound (gallop rhythm), a positive hepatojugular reflux sign, elevated jugular venous pressure, and cardiac apical impulse displacement to the left and downward. Regarding biomarkers, an abnormal diagnosis is suggested by plasma BNP levels exceeding 35 pg/mL or N-terminal pro-B-type natriuretic peptide (NT-proBNP) levels surpassing 125 pg/mL. Furthermore, objective evidence of structural or functional cardiac alterations is obligatory to substantiate the diagnosis. For cases presenting with diagnostic uncertainty, definitive confirmation of heart failure may necessitate stress testing or invasive hemodynamic evaluation (e.g., direct measurement of left ventricular filling pressure)^17^.

#### Exclusion indicators

Missing hs-CRP measurement data at admission (*n* = 1,067).

### Data extraction

Baseline characteristics including gender, age, heart failure classification, temperature, pulse, blood pressure measurements, BMI, and CCI score were recorded. On the first day of hospital admission, after patients had fasted for eight hours, laboratory tests were carried out. The parameters measured included serum uric acid, creatinine, cystatin C, D - dimer, high - sensitivity troponin, brain natriuretic peptide (BNP), glutamic - oxaloacetic transaminase, gamma - glutamyl transferase, total cholesterol, triglycerides, albumin, potassium, and sodium.

### Endpoints and covariates

After patients were discharged, follow - up data were gathered, mainly concentrating on readmission and mortality rates. When patients could not come to the clinical center, follow - up evaluations were carried out by phone^17^. In the initial database, the main outcome variable was all - cause mortality within six months. These outcome variables were noted as dichotomous, with survival marked as 0 and death marked as 1.

### Statistical analysis

SPSS 27.0 software was employed to perform statistical analyses. The normality of continuous variables was evaluated by the Kolmogorov - Smirnov test. For variables with a normal distribution, data were shown as means ± standard deviation (SD), and two sample independent t-test were utilized for group comparisons. Natural logarithm transformations were applied to hs-CRP, BNP, ALT, and hs-TnT due to their skewed distributions and residuals, thereby normalizing data and stabilizing variance. Categorical variables were presented in the form of frequencies and percentages. Group differences were examined through χ² tests for comparison purposes. One-way ANOVA was employed to analyze differences in continuous variables across the three groups. Potential confounders were identified based on prior knowledge and univariate correlation with the primary endpoint. Covariates in the multivariate binary logistic regression analysis consisted of variables that demonstrated statistical significance (*P* < 0.1). To address multicollinearity, variance inflation factor (VIF) screening was performed, and variables with VIF values exceeding five were excluded^19^. In order to assess the relationship that exists between hs - CRP and all - cause mortality in HF patients, binary logistic regression analysis was conducted using three progressive adjustment models: Model I (unadjusted), Model II (age and sex adjusted), and Model III (further adjusted for creatinine, uric acid, cystatin C, log-transformed BNP, log-transformed high-sensitivity troponin T, and potassium). The predictive performance of hs-CRP for all-cause mortality was estimated using receiver operating characteristic (ROC) curve analysis with calculation of area under the curve (AUC). The power analysis was conducted using G*Power software (version 3.1.97; available at http://www.gpower.hhu.de/ ). Statistical significance was set at a two - tailed P value less than 0.05.

## Result

### Baseline characteristics of patients

We identified 1,067 individuals lacking hs-CRP data, and performed inter-group comparisons, comparing included (*n* = 941) and excluded (*n* = 1,067) patients showed that included patients had higher CCI score (1.96 ± 0.98 vs. 1.78 ± 0.94, *p* < 0.001), elevated D-dimer levels and decreased levels of Uric acid level. (Table 1)

**Table 1:** Baseline characteristics of patients with hs-CRP group and no hs-CRP group.

Variables	Hs-CRP group	No hs-CRP group	t/χ^2^	*P*
No. of patients	941	1067		
Age (year)			0.483	0.486
≥ 60	862 (91.6%)	968 (90.7%)		
21–59	79 (9.2%)	99 (9.3%)		
Sex			0.716	0.717
Male	400 (42.5%)	445 (41.7%)		
Female	541 (57.5%)	622 (58.3%)		
Body temperature (℃)	36.43 ± 0.45	36.40 ± 0.42	1.689	0.091
Pulse (bpm)	86.46 ± 20.78	84.15 ± 22.15	2.396	0.057
DBP (mmHg)	76.33 ± 14.28	76.79 ± 14.63	– 0.71	0.476
BMI (kg/m^2^)	21.29 ± 3.88	21.31 ± 3.75	– 0.14	0.889
CCI score	1.96 ± 0.98	1.78 ± 0.94	4.095	< 0.001
LVEF (%)	50.58 ± 13.27	50.82 ± 13.09	– 2.236	0.813
Creatinine (µmol/L)	9.36 ± 5.39	9.75 ± 5.68	– 1.559	0.119
Uric acid (µmol/L)	472.04 ± 165.0	492.84 ± 173.2	– 2.732	0.006
Cystatin (mg/l)	1.85 ± 0.94	1.84 ± 0.96	0.104	0.917
D-dimer (mg/L)	2.85 ± 6.29	2.08 ± 4.27	3.102	0.002
Albumin (g/L)	36.22 ± 4.98	36.80 ± 4.96	– 2.528	0.052
Log(ALT) (U/L)	1.62 ± 0.37	1.61 ± 0.36	0.388	0.698
Log(BNP) (pg/ml)	2.84 ± 0.55	2.83 ± 0.53	0.159	0.874
Log(hs-TnT) (pg/ml)	1.93 ± 0.95	2.02 ± 0.99	0.816	0.366
Triglyceride (mmol/l)	1.14 ± 1.06	1.18 ± 1.10	– 0.860	0.390
TC (mmol/l)	3.73 ± 1.09	3.71 ± 1.09	0.391	0.696
potassium (mmol/l)	3.95 ± 0.71	4.01 ± 0.70	– 2.019	0.054
Sodium (mmol/l)	138.18 ± 5.00	138.27 ± 4.82	– 0.393	0.694

*DPB* diastolic blood pressure, *BMI* body mass index, *CCI score* Charlson Comorbidity Index score, *LVEF* Left ventricular ejection fractions, *ALT* glutamyl transpeptidase, *BNP* Brain Natriuretic Peptide, *hs-TnT* highly sensitive troponin, *TC* total cholesterol.

The retrospective cohort research enrolled 941 HF patients. The 6-month HF mortality rate was 3.08% (29/941). After stratification by log-transformed hs-CRP (log(hs-CRP)) tertiles, mortality rates were 1.90% (6/316), 1.92% (6/313), and 5.45% (17/312) across ascending tertiles, respectively (Table 2).

**Table 2:** Baseline characteristics of patients with heart failure grouped by log(hs-CRP) tertile.

Variables	Tertile Log (hs-CRP) (mg/L)			t/χ^2^	*P*
	T1 (≤ 0.71)	T2 (0.71–1.28)	T3 (≥ 1.28)		
No. of patients	316	313	312		
Age (years)				9.648	0.008
≥ 60	292 (92.4%)	296 (94.6%)	274 (87.8%)		
21–59	24 (7.6%)	17 (5.4%)	38 (12.2%)		
Sex				19.172	< 0.001
Male	103 (32.6%)	150 (47.9%)	147 (47.1%)		
Female	213 (67.4%)	163 (52.1%)	165 (52.9%)		
Body temperature (℃)	36.34 ± 0.30	36.43 ± 0.47	36.54 ± 0.55	15.412	< 0.001
Pulse (bpm)	82.10 ± 20.96	87.27 ± 19.76	90.07 ± 20.85	12.189	< 0.001
DBP (mmHg)	77.57 ± 13.66	77.38 ± 14.91	74.02 ± 14.00	6.187	0.002
BMI (kg/m^2^)	21.41 ± 3.82	21.47 ± 4.11	20.99 ± 3.69	1.404	0.246
CCI score	1.90 ± 0.91	1.89 ± 0.97	2.08 ± 1.06	3.622	0.027
LVEF (%)	52.26 ± 13.13	49.54 ± 13.96	49.52 ± 12.92	1.527	0.219
Creatinine (µmol/L)	8.54 ± 4.64	9.02 ± 4.90	10.53 ± 6.31	11.819	< 0.001
Uric acid (µmol/L)	442.91 ± 148.91	482.97 ± 160.51	490.48 ± 180.69	7.627	0.001
Cystatin (mg/l)	1.84 ± 0.95	1.85 ± 0.96	1.78 ± 0.89	13.476	< 0.001
D-dimer (mg/L)	2.02 ± 5.67	2.77 ± 6.65	3.78 ± 6.41	5.753	0.003
Albumin (g/L)	37.77 ± 4.50	36.82 ± 4.43	34.06 ± 5.22	50.350	< 0.001
Log(ALT) (U/L)	1.56 ± 0.34	1.61 ± 0.38	1.70 ± 0.39	10.587	< 0.001
Log(BNP) (pg/ml)	2.74 ± 0.55	2.86 ± 0.55	2.91 ± 0.53	8.316	< 0.001
Log(hs-TnT) (pg/ml)	1.79 ± 1.04	1.51 ± 0.95	1.63 ± 0.85	1.043	0.936
Triglyceride (mmol/l)	1.15 ± 0.69	1.13 ± 1.49	1.13 ± 0.80	0.014	0.986
TC (mmol/l)	3.82 ± 1.10	3.76 ± 1.05	3.62 ± 1.12	2.667	0.070
potassium (mmol/l)	3.91 ± 0.57	3.90 ± 0.79	4.03 ± 0.75	2.933	0.054
Sodium (mmol/l)	139.80 ± 3.91	138.78 ± 4.86	135.94 ± 5.31	55.927	< 0.001

*DPB* diastolic blood pressure, *BMI* body mass index, *CCI score* Charlson Comorbidity Index score, *LVEF* Left ventricular ejection fractions, *ALT* glutamyl transpeptidase, *BNP* Brain Natriuretic Peptide, *hs-TnT* highly sensitive troponin, *TC* total cholesterol.

Baseline clinical characteristics (Table 2) showed no significant intergroup differences for body mass index (BMI), left ventricular ejection fraction (LVEF), log(hs-TnT), triglycerides, total cholesterol, or serum potassium levels (all *P* > 0.05). However, significant differences emerged for age, sex, vital signs (body temperature, diastolic blood pressure, heart rate), comorbidity burden (Charlson Comorbidity Index score), renal function markers (serum creatinine, uric acid), inflammatory markers (cystatin, D-dimer), nutritional status (albumin), and log(BNP) and log(ALT) (all *P* < 0.05).

### Univariate analysis

Single variable analysis (Table 3) demonstrated a significant positive association between admission-based log(hs-CRP) levels and all-cause mortality during the 6-month follow-up period (OR = 2.717, 95% CI: 1.414–5.219, *P* = 0.003). Additional significant predictors included elevated serum creatinine, hyperuricemia, increased cystatin, potassium levels, and elevated log(BNP) and log(hs-TnT).

**Table 3:** Univariate analysis for all cause of death during 6 months after discharge.

Variables	Statistics	OR	95% CI	*P*
Log (hs-CRP)	1.02 ± 0.61	2.717	1.414–5.219	0.003
Age ≥ 60 year	1830 (91.14%)	1.012	0.399–2.567	0.098
Sex (female)	1163 (57.92%)	1.441	0.850–2.440	0.175
Body temperature (℃)	36.43 ± 0.46	1.650	0.864–3.150	0.129
Pulse (bpm)	86.46 ± 20.78	1.009	0.992–1.026	0.289
DBP (mmHg)	76.33 ± 14.28	0.987	0.961–1.013	0.330
BMI (kg/m^2^)	21.29 ± 3.88	0.985	0.894–1.086	0.767
CCI score	1.96 ± 0.98	1.248	0.871–1.786	0.227
LVEF (%)	50.54 ± 13.38	1.001	0.930–1.078	0.975
Creatinine (µmol/L)	9.57 ± 5.55	1.127	1.078–1.178	< 0.001
Uric acid (µmol/L)	472.04 ± 165.0	1.002	1.000-1.004	0.017
Cystatin (mg/l)	1.85 ± 0.94	1.797	1.375–2.349	< 0.001
D-dimer (mg/L)	2.85 ± 6.29	1.028	0.999–1.058	0.054
Albumin (g/L)	36.22 ± 4.98	0.936	0.870–1.007	0.075
Log(ALT) (U/L)	1.62 ± 0.37	0.782	0.278–2.195	0.640
Log(BNP) (pg/ml)	2.84 ± 0.55	1.000	1.000-1.001	< 0.001
Log(hs-TnT) (pg/ml)	2.71 ± 3.41	0.824	0.695–0.977	0.026
Triglyceride(mmol/l)	1.14 ± 1.06	0.953	0.589–1.544	0.846
TC (mmol/l)	3.73 ± 1.09	0.931	0.644–1.346	0.703
potassium (mmol/l)	3.95 ± 0.71	1.599	1.110–2.302	0.012
Sodium (mmol/l)	138.18 ± 5.00	0.960	0.900-1.025	0.220

*DPB* diastolic blood pressure, *BMI* body mass index, *CCI score* Charlson Comorbidity Index score, *LVEF* Left ventricular ejection fractions, *ALT* glutamyl transpeptidase, *BNP* Brain Natriuretic Peptide, *hs-TnT* highly sensitive troponin, *TC* total cholesterol.

### Association between log(hs-CRP) and mortality

After adjusting for potential confounders through multivariate modeling, logistic regression analysis (Table 4) confirmed a significant association between elevated log(hs-CRP) concentrations and increased mortality risk (OR = 2.073, 95% CI: 1.009–4.256, *P* = 0.047). When log(hs-CRP) concentrations were categorized into tertiles, this association was attenuated. Following comprehensive covariate adjustment, elevated CRP levels exhibited a positive correlation with increased mortality risk. However, the relationship did not achieve statistical significance in the multivariable model (OR = 1.739, 95% CI: 0.632–4.790, *P* = 0.199).

**Table 4:** Relationship between log(hs-CRP) and all cause of death during 6 months in different models.

Variable	Model 1		Model 2		Model 3	
	OR (95% CI)	*P*-value	OR (95% CI)	*P*-value	OR (95% CI)	*P*-value
Log(hs-CRP)	2.717 (1.414–5.219)	0.003	2.706 (1.397–5.239)	0.003	2.073 (1.009–4.256)	0.047
CRP tertile						
Q1 group	Reference	–	Reference	–	Reference	–
Q2 group	1.010 (0.322–3.165)	0.987	0.921 (0.291–2.910)	0.888	0.745 (0.225–2.467)	0.630
Q3 group	2.977 (1.158–7.655)	0.024	2.856 (1.100-7.418)	0.031	1.739 (0.632–4.790)	0.284
P for trend	0.018		0.018		0.199	

*CI* confidence interval, Model 1 adjusted for none; Model 2 adjusted for age, sex; Model 3 adjusted for age, sex, Creatinine, Uric acid, Cystatin, Log(BNP), Log(hs-TnT) and potassium.

**Fig. 1:**
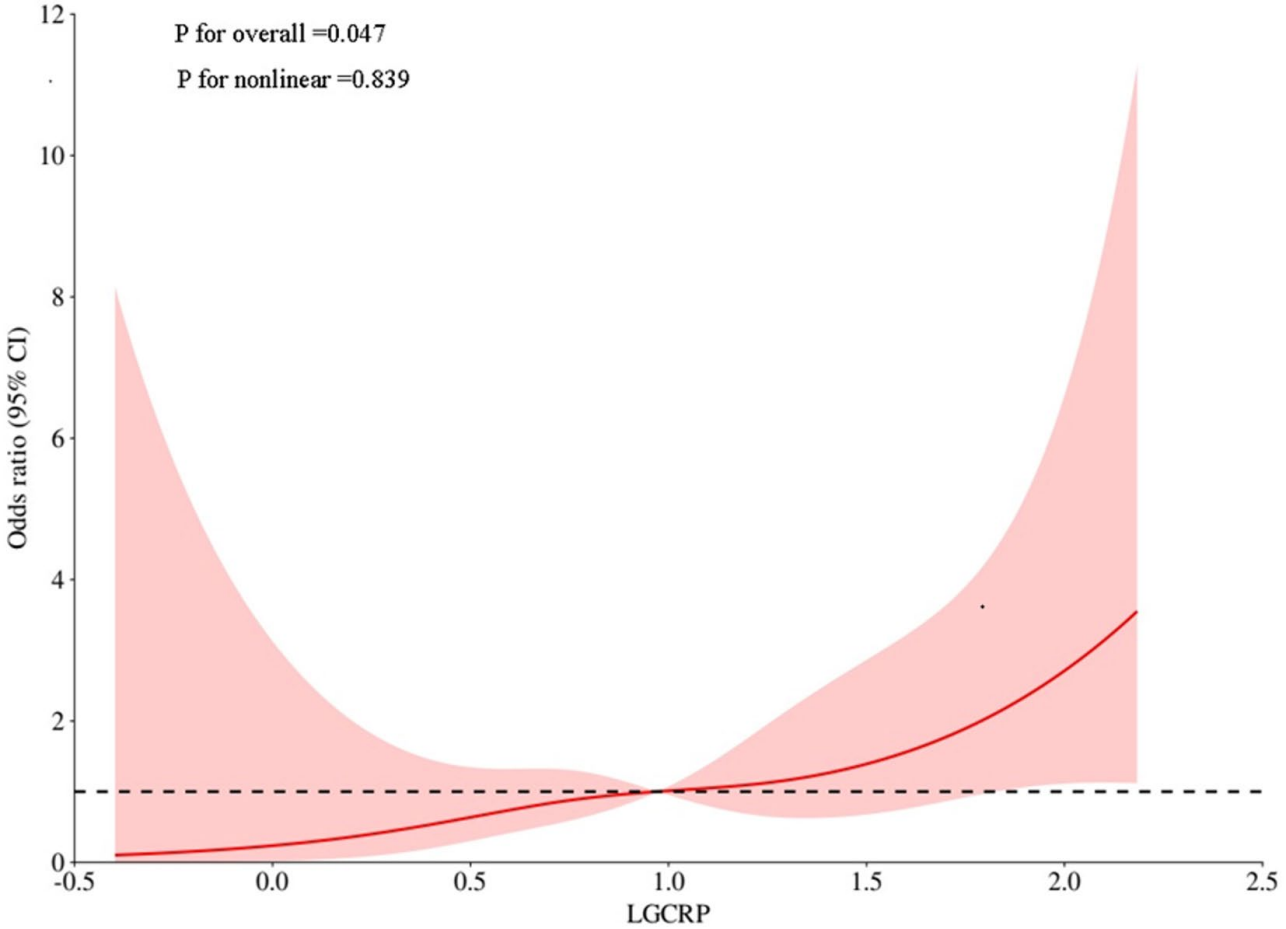
The correlation between log(hs-CRP) levels and mortality risk.

We examined the potential non-linear correlation between log(hs-CRP) levels and mortality risk (Fig. 1) using log likelihood ratio testing (*P* = 0.839), which effectively excluded non-linear associations. These findings suggest that log(hs-CRP) elevation generally correlates with increased mortality risk, warranting cautious interpretation of clinical log(hs-CRP) cutoff values, particularly in populations with markedly elevated inflammatory markers.

### Predictive ability of log(hs-CRP) for 6-months of all-cause mortality

**Fig. 2:**
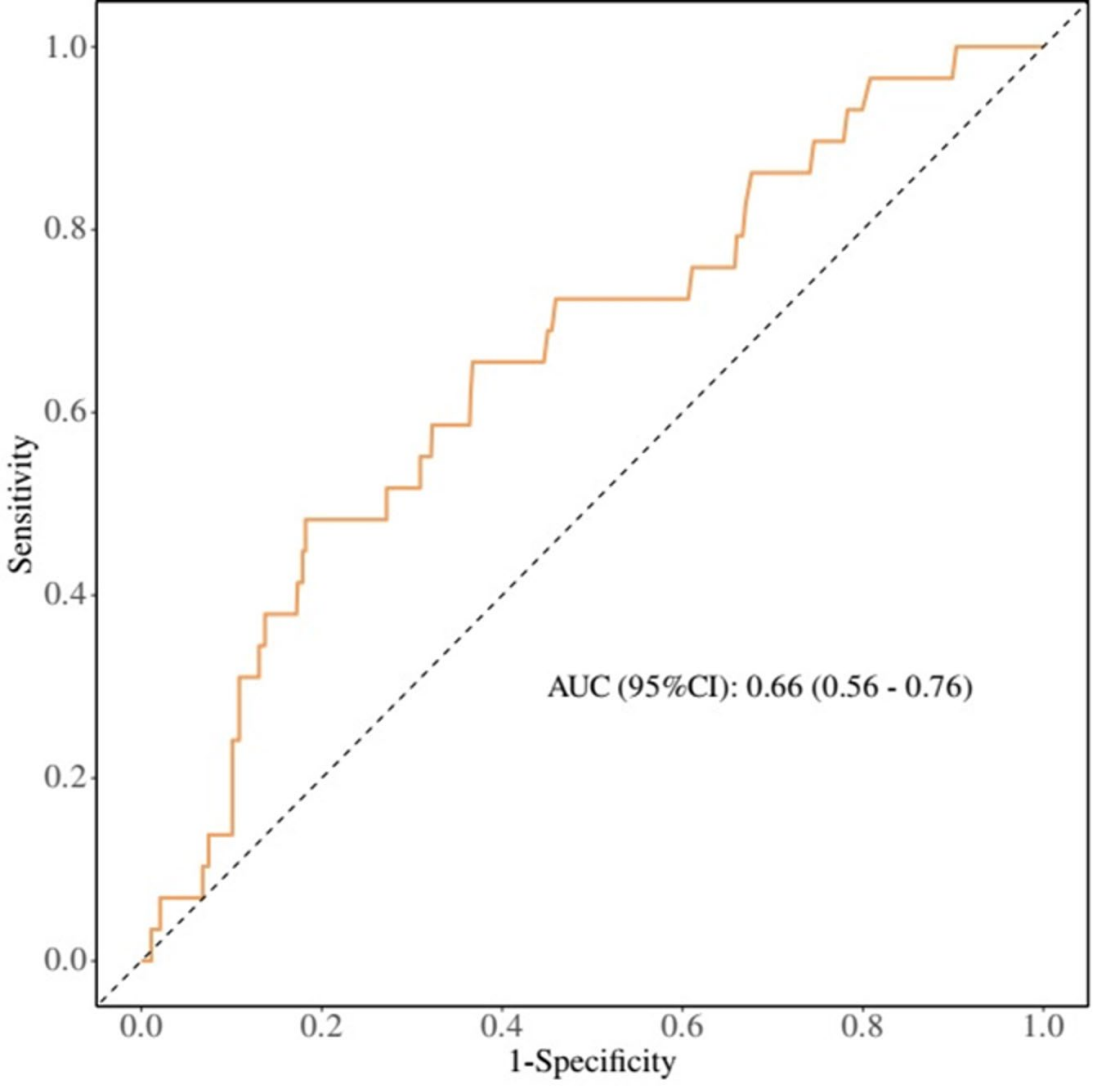
Predictive ability of log(hs-CRP) for 6-months of all-cause mortality.

As shown in Fig. 2, receiver operating characteristic curve analysis demonstrated that log(hs-CRP) has modest ability to predict mortality (AUC: 0.66, 95% CI: 0.56–0.76). Diagnostic performance parameters revealed a sensitivity of 0.82 (95% CI: 0.79–0.84) and specificity of 0.48 (95% CI: 0.30–0.66), with a cutoff value of 1.64. While the model demonstrates predictive accuracy superior to random chance, its overall efficacy remains suboptimal.

## Discussion

Among a sizeable retrospective cohort consisting of Chinese patients with HF, we studied the relationship between baseline log(hs-CRP) and mortality. This study evaluated the association between log(hs-CRP) and HF outcomes by analyzing all-cause mortality within six months as a criterion for assessing long-term prognosis. After adjusting for potential confounding factors- including age, sex, creatinine, uric acid, cystatin, BNP, hs-TnT and potassium—binary logistic regression analysis demonstrated that log(hs-CRP) is an independent risk factor for all-cause mortality in patients with HF. ROC curve analysis confirmed that log(hs-CRP) has significant predictive value for HF prognosis. These findings highlight the importance of hs-CRP levels in predicting long-term outcomes among with HF patients.

The association between hs-CRP and HF has been extensively studied. Data from early investigations indicate that hs-CRP positively correlates with heart failure severity and adverse prognostic risk^5,8,20,21^. Although existing literature has predominantly focused on Western populations, limited investigations have specifically addressed hs-CRP-mortality associations within Chinese demographics. Our research showed a consistent association between elevated log(hs-CRP) concentrations and increased all-cause mortality risk. Furthermore, our current investigation extends beyond these findings by conducting a comprehensive ROC curve analysis. Notably, this study represents the first systematic identification of an inflection point and quantitative characterization of the threshold effect in the hs-CRP-heart failure relationship among Chinese adults.

HF is a life-threatening syndrome resulting from congenital or acquired cardiac structural or functional abnormalities, often accompanied by elevated levels of proinflammatory cytokines and chemokines^6^. Substantial evidence indicates that persistent inflammatory mediators contribute to disease onset, progression, and complications^7^. Elevated levels of these inflammatory markers have emerged as independent prognostic indicators in HF, correlating with adverse clinical outcomes^22^. Hs-CRP plays a multifaceted part in atherogenesis by enhancing the uptake and oxidation of low-density lipoprotein (LDL), inhibiting nitric oxide (NO) production, upregulating the expression of adhesion molecules, and inhibiting fibrinolysis through escalated expression of plasminogen activator inhibitor-1 (PAI-1). Additionally, hs-CRP induces complement activation and promotes monocyte infiltration into the vascular wall^23,24^. These pathophysiological processes collectively contribute to accelerated progression of cardiovascular disorders, precipitation of congestive heart failure events, and ultimately portend an unfavorable prognostic trajectory. Consistent with previous studies, our findings demonstrate that higher log(hs-CRP) levels are associated with an increased likelihood of mortality in patients with heart failure^8–12,25,26^. These results suggest that hs-CRP can be utilized as a crucial biological marker for predicting both the occurrence of heart failure and long-term clinical outcomes.

Current evidence suggests that hs-CRP does not function as a medium or signaling molecule within inflammatory cascades, thereby limiting its therapeutic utility as a direct target for inflammatory intervention^27^. Nevertheless, accumulating data confirm that various anti-inflammatory pharmacological agents achieve clinically meaningful reductions in CRP levels^28–31^. Of particular significance, established cardiovascular therapies including statins, ACEI and β-adrenergic receptor antagonists, have demonstrated robust CRP-lowering effects in clinical settings^32–34^. Preclinical investigations further confirm the therapeutic potential of engineered agents specifically designed to neutralize CRP activity^35^. Crucially, while these observations suggest a potential link between hs-CRP modulation and cardiovascular outcomes, the prognostic significance of hs-CRP reduction in heart failure remains unresolved regarding its impact on mortality reduction. This knowledge gap underscores the imperative for large-scale randomized controlled trials to elucidate whether hs-CRP serves merely as a surrogate biomarker reflecting underlying pathophysiological mechanisms or constitutes a modifiable risk factor where targeted hs-CRP reduction could confer tangible clinical benefits in heart failure management.

Our analysis revealed a linear relationship between log(hs-CRP) and 6-month mortality, rather than a non - linear relationship (log-rank *p* = 0.839), supporting proportional risk stratification using either continuous values or the identified 1.64 cutoff (AUC = 0.66). Each unit increase in log(hs-CRP) conferred a 2.07-fold mortality risk (adjusted OR = 2.073, *p* = 0.047). The optimal hs-CRP cutoff (1.64) demonstrated 82% sensitivity and 48% specificity for predicting 6-month mortality in HF patients, offering a practical tool for risk stratification [Figure 2]. Unlike BNP and troponin, which reflect myocardial stress and injury, hs-CRP captures systemic inflammation—a complementary pathway in HF progression^6,7^. Multivariate analysis confirmed its independent prognostic value (OR = 2.073, *p* = 0.047) even after adjusting for log(BNP) and log(hs-TnT) (Table 4). While hs-CRP should augment rather than replace established biomarkers, its integration may improve risk stratification by quantifying inflammation-driven HF progression^7,22^. Further validation in multicenter cohorts is needed to refine its role in composite risk scores.

This cohort study examined the association between hs-CRP levels and 6-month mortality in a population of 941 patients, observing an overall mortality rate of 3.08% (29 deaths). Stratification by the predefined hs-CRP cutoff of 1.64 revealed significant differences: the high hs-CRP group (> 1.64, *n* = 180) demonstrated substantially higher mortality (7.8%, 14 deaths) compared to the low hs-CRP group (≤ 1.64, *n* = 761; 1.97%, 15 deaths). Using G*Power software with a two-sided α of 0.05, we calculated a statistical power of 98.5%, indicating robust capability to detect this clinically meaningful association between elevated hs-CRP levels and increased 6-month mortality risk.

In this investigation, multivariate logistic regression analysis and curve fitting techniques were applied to establish an initial linear positive relationship between hs-CRP levels and six-month mortality in HF patients. However, our research has several limitations. First, a notable limitation of this study is the lack of detailed data on in-hospital medications (statins, ACEIs, β-blockers) from the retrospective use of existing clinical registries. Despite standardized treatment at a single institution reducing therapeutic variability, we could not quantify how these inflammation-modulating drugs—known to affect hs-CRP—influence biomarkers or mortality. This unadjusted confounding factor may have biased the observed association between hs-CRP and 6-month mortality. The future prospective studies with systematic medication data collection could better define hs-CRP’s independent prognostic value. Second, the single-center design in Sichuan Province may limit the generalizability of our findings to the broader Chinese population, as regional differences in healthcare resources and patient characteristics could affect results. Future multicenter studies are warranted to validate these findings across diverse Chinese populations. Third, even though multiple covariates were adjusted for within the regression model, there could still be confounding variables that are either unknown or cannot be identified. For example, the source data did not record dynamic changes in CRP levels, leaving the prognostic significance of sustained elevations in hs-CRP incompletely evaluated. Fourth, the follow-up period of 6 months is relatively short, with a correspondingly small number of reported positive outcomes (deaths), necessitating extended follow-up durations and enrollment of larger cohorts for more robust clinical evaluation. Finally, a key limitation of this study was the exclusion of 1,067 patients due to missing hs-CRP data, which may have introduced selection bias. Although baseline markers of heart failure severity did not differ significantly between the excluded and included cohorts, the excluded group exhibited a higher comorbidity burden. These factors could constrain the generalizability of the findings in future applications.

## Conclusion

This study identified a remarkable and independent positive relationship between admission log(hs-CRP) levels and all-cause mortality within six months, even after adjusting for other covariates. Additionally, our analysis uncovered a linear relationship between log(hs-CRP) levels and mortality. Moreover, the identified cut-off points may hold clinical significance for risk stratification, offering a potential complementary tool to existing biomarkers. These results highlight the potential importance of inflammatory markers in evaluating the long-term prognosis of patients with heart failure.

## Data Availability

The datasets used and/or analysed during the current study available from the corresponding author on reasonable request.
